# *Alocasia sakonakhonensis* (Araceae), a new species from northeastern Thailand

**DOI:** 10.1002/ece3.11462

**Published:** 2024-05-24

**Authors:** Wilawan Promprom, Phukphon Munglue, Wannachai Chatan

**Affiliations:** ^1^ Department of Biology, Faculty of Science Mahasarakham University Maha Sarakham Thailand; ^2^ Plant and Innovation Research Unit Mahasarakham University Maha Sarakham Thailand; ^3^ Program of Biology, Faculty of Science Ubon Ratchathani Rajabhat University Nai Mueang Thailand

**Keywords:** aroid, Aroideae, biodiversity, flowering plant, monocots, new taxa

## Abstract

*Alocasia sakonakhonensis* Chatan & Promprom (Araceae), a new species from northeastern Thailand, is described and illustrated. It is clearly different from other previously known species by leaves, spathe, ovary, sterile interstice, synandria, and appendix. Color illustrations, and a distribution map are provided, as well as comparative morphological characters about its similar species. The preliminary conservation status assessment of the new species was presented.

## Graphical abstract

*Alocasia sakonakhonensis* Chatan & Promprom (Araceae), a new species from Thailand, is described and illustrated. It is clearly different from other previously known species by leaves, spathe, ovary, sterile interstice, synandria, and appendix. The preliminary conservation status assessment of the new species was considered as “Endangered (EN),” according to the Guidelines for using the IUCN red list categories and criteria. Version 16 (IUCN Standards and Petitions Committee, 2024).

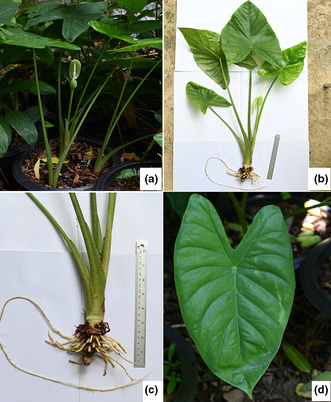

## 1 INTRODUCTION

*Alocasia* Schott is a genus in the family Araceae, includes about 100 species of diminutive to massive pachycaul‐arborescent terrestrial or epilithic mesophytes (rather rarely–helophytes), occurring from Sri Lanka and India, throughout Indochina to China and southern Japan, the Malesian Archipelago, Australia and Oceania (Hay, 1999; Nauheimer et al., 2012; POWO, 2024; Zulhazman et al., 2017), and its species are usually evergreen or very rarely seasonally dormant (Boyce & Sookchaloem, 2012).

In Thailand, Boyce (2008) reported the discovery of nine species, Boyce and Sookchaloem (2012) recognized 10 described species, including one endemic species (*A. hypoleuca* P.C.Boyce), and the most recently found in Thailand (*A. epilithica* Serebryanyi, K.Z.Hein & Naïve) (Hein et al., 2022).

During our floristic surveys in the Northeast of Thailand done by the authors in 2019–2022, many exciting specimens belonging to the family Araceae were found. Also, *Alocasia* specimens were collected to study their morphology. After carefully studying their morphological characters, the authors found that they were not matched to other previously known *Alocasia* species, representing an undescribed species. We describe and illustrate another new *Alocasia* species from this area in the present paper, namely *Alocasia sakonakhonensis* Chatan & Promprom.

## 2 MATERIALS AND METHODS

Field trips were conducted in the Phu Phan mountain range located in Sakon Nakhon province, situated in the Northeast of Thailand, spanning from 2019 to 2022. During these trips, plant specimens were meticulously collected for the purpose of study. The type specimens and herbarium specimens belonging to morphologically related species within the *Alocasia* genus (obtained from Royal Botanic Gardens, Kew (K)), Forest Herbarium, Department of National Parks, Wildlife and Plant Conservation (BKF) and Bangkok Herbarium, Plant Varieties Protection Office (BK) were meticulously examined. Additionally, digitized images of specimens available on the JSTOR Global Plant database (https://plants.jstor.org/) were also analyzed.

In order to enhance our understanding, pertinent references (such as Boyce, 2007; Boyce, 2008; Boyce & Sookchaloem, 2012; Hay & Wise, 1991; Hay, 1996; Hay, 1998; Hay, 1999; Li & Boyce, 2010; Nicolson, 1987, etc.) were consulted. Morphological measurements were conducted on fully developed specimens of mature flowering plants in their natural habitat. This was supplemented by measurements taken from herbarium specimens.

Upon a comprehensive examination of these specimens, they revealed characteristics that did not align with any previously documented taxon. Subsequently, a new *Alocasia* species was identified and is herein described and illustrated. Detailed morphological descriptions and a species key pertinent to *Alocasia* in Thailand are provided. The terminology concerning plant attributes adheres to the conventions laid out by Boyce (2008) and Boyce and Sookchaloem (2012). Furthermore, an evaluation of conservation status was executed in accordance with the Guidelines for Using the IUCN Red List Categories and Criteria, Version 16 (IUCN Standards and Petitions Committee, 2024).

## 3 TAXONOMIC TREATMENT

*Alocasia sakonakhonensis* chatan & promprom, sp. nov.

### 3.1 Type

**FIGURE 1:**
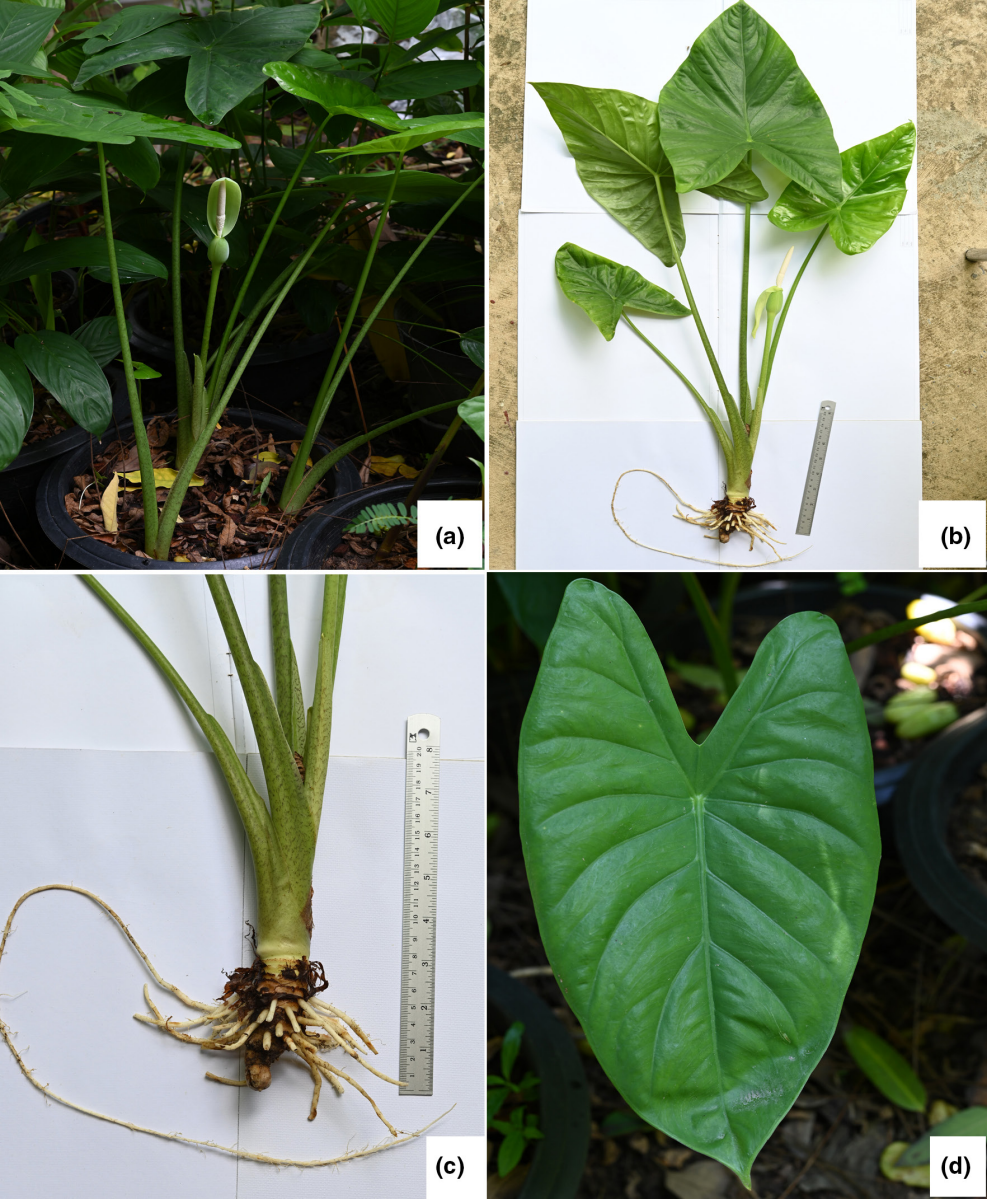
*Alocasia sakonakhonensis* Chatan & Promprom (a). habit; (b). whole plant; (c). rhizome and leaf sheath; (d). leaf blade. All are photographed by Wilawan Promprom.

**FIGURE 2:**
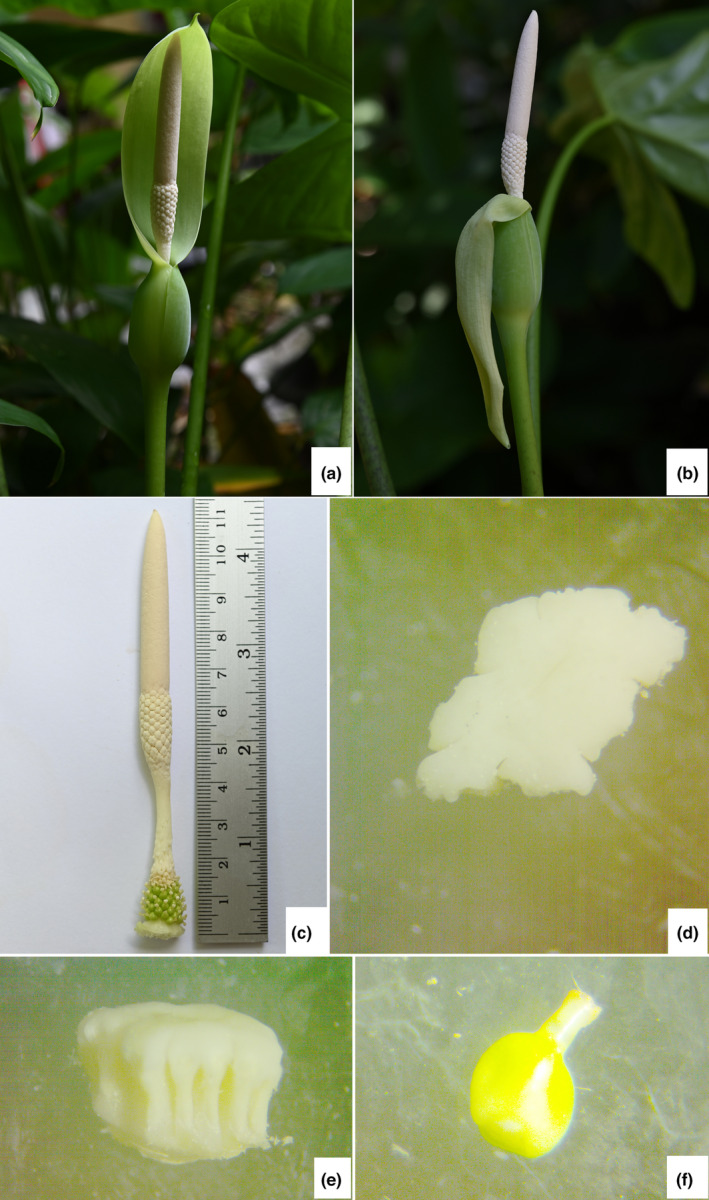
*Alocasia sakonakhonensis* Chatan & Promprom (a). inflorescence before the spathe limb bending downward; (b). inflorescence during the spathe limb bending downward; (c). spadix; (d). synandria (top view); (e). synandria (lateral view); (f). pistillate flower. All are photographed by Wilawan Promprom.

Thailand. Sakon Nakhan Province: Khok Sri Suphan District, 300–380 m elev., ''16°59'45.5'' N 104°15'46.9'' E, June 20, 2019, *W. Chatan 2591* (holotype: BKF!; isotype: BK!). Figures 1 and 2.

### 3.2 Diagnosis

*Alocasia sakonakhonensis* Chatan & Promprom is most similar to *Alocasia acuminata* Schott (Boyce, 2008; Boyce & Sookchaloem, 2012), but the former is readily distinguishable by the presence of moderately green with numerous dirty mottled dark brown 1–3 mm lines or areas (vs bright green without these dirty mottled dark brown 1–3 mm lines or areas); longer peduncle, spathe, spathe limb, spadix, sterile interstice, and appendix; mostly distinctly 2–4 lobed or rarely unlobed stigma (vs unlobed or very slightly lobed stigma; opened petiolar sheath (vs closed in *A. acuminata*); dull yellow‐brown appendix (vs white one found in *A. acuminata*).

Small to medium‐sized, slightly robust, evergreen terrestrial herbs, up to 60 cm tall. Stem rhizomatous, generally elongate, ca. 3–8 cm long and 2.8–3.1 cm diameter, erect and short, inside yellowish‐green, older parts covered with remains of old leaf bases and cataphylls; cataphylls 6–8 × 2.5–3.3 cm, green with dirty dark brown spots, glabrous, keel near apex, apex retuse with a small projection, margin entire and translucent. Roots numerous, white. Leaves 2–4 together, subtended by conspicuous lanceolate papery‐membranous cataphylls; Cataphylls 14–15 × 1.5–1.8 cm, moderately green with numerous dirty mottled dark brown 1–3 mm lines or areas, glabrous, translucent. Petioles glabrous, moderately green with numerous dirty mottled dark brown 1–3 mm lines or areas, ca. 35–60 cm long (i.e., including sheathing in the lower part ca. 14–18.), sheath closed; lamina spreading, ovate, 20–40 × 14–22 cm, bright green, base slightly hastate to hastate, peltate for 37%–43% of their length; posterior lobes 10–17.5 cm long, apex obtuse; anterior lobe 13–26 cm long, apex cuspidate; anterior costa with 5–7 primary lateral veins on each side, the proximal ones diverging at ca. (50°–) 60–80°, the angle decreasing in distal veins and the course more or less straight to the margin; axillary glands hardly conspicuous abaxially; secondary venation initially wide‐spreading, then sooner or later deflected toward the margin; interprimary collecting veins weakly defined. Inflorescences usually solitary. Peduncles 25–30 cm long, moderately green with numerous dirty mottled dark brown 1–3 mm lines or areas, erect at first, then declinate, elongating and then erect in advanced fruit, subtended by 1–2 cataphylls; the cataphylls linear‐lanceolate, 16.5–18.5 × 2.5–2.6 cm, keel near apex, apiculate apex, translucent. Spathe 13.0–14.5 × 3.0 cm, moderately constricted ca. 3.5–4.0 cm from the base; lower part of spathe bright green, ovoid; limb lanceolate, canoe‐shaped and longitudinally hooded, 9.5–10.5 × 2.3–2.5 cm, apex apiculate, membranaceous, very pale green. Spadix shorter than the spathe, ca. 11–12 cm long, sessile; female flower zone 1.0–1.2 cm; ovary globose, slightly 3‐lobed‐globose or depressed globose or slightly elliptic, 2–3 mm diam., light green; style 1.2–1.5 mm long; stigma off‐white with a hint of yellow or strong pale yellow, distinctly 2–4 lobed or rarely unlobed; sterile interstice subcylindrical, 23–25 mm, narrower than the fertile zones, corresponding with the spathe constriction, off‐white with a hint of yellow or strong pale yellow; lower and upper synandrodia often with incompletely connate staminodes, flat‐topped; male flower zone subcylindric, 2.0–2.3 × 6–7 mm, off‐white with a hint of yellow or strong pale yellow; synandria 4–8‐merous, more or less hexagonal to circular, 1.5–4.0 mm diam.; appendix 5.0–5.2 cm long, slightly narrower than male flower zone, cylindrical lower part ca. 2/3, elongate‐conic upper part ca. 1/3, dull yellow‐brown. Fruiting spathe ovoid, ca. 14 cm long, green. Fruits globose‐ellipsoid, ca. 0.7–0.8 cm diameter, green, ripened orange‐red.

### 3.3 Additional specimens examined

*Alocasia sakonakhonensis* Chatan & Promprom: THAILAND. Sakon Nakhon Province: Phu Phan mountain range, 300–320 m alt., June 15, 2020, *W. Chatan 2592* (Paratype: BKF).

### 3.4 Phenology

The flowering period is from May to June, and the fruiting period is June to July.

### 3.5 Distribution

**FIGURE 3:**
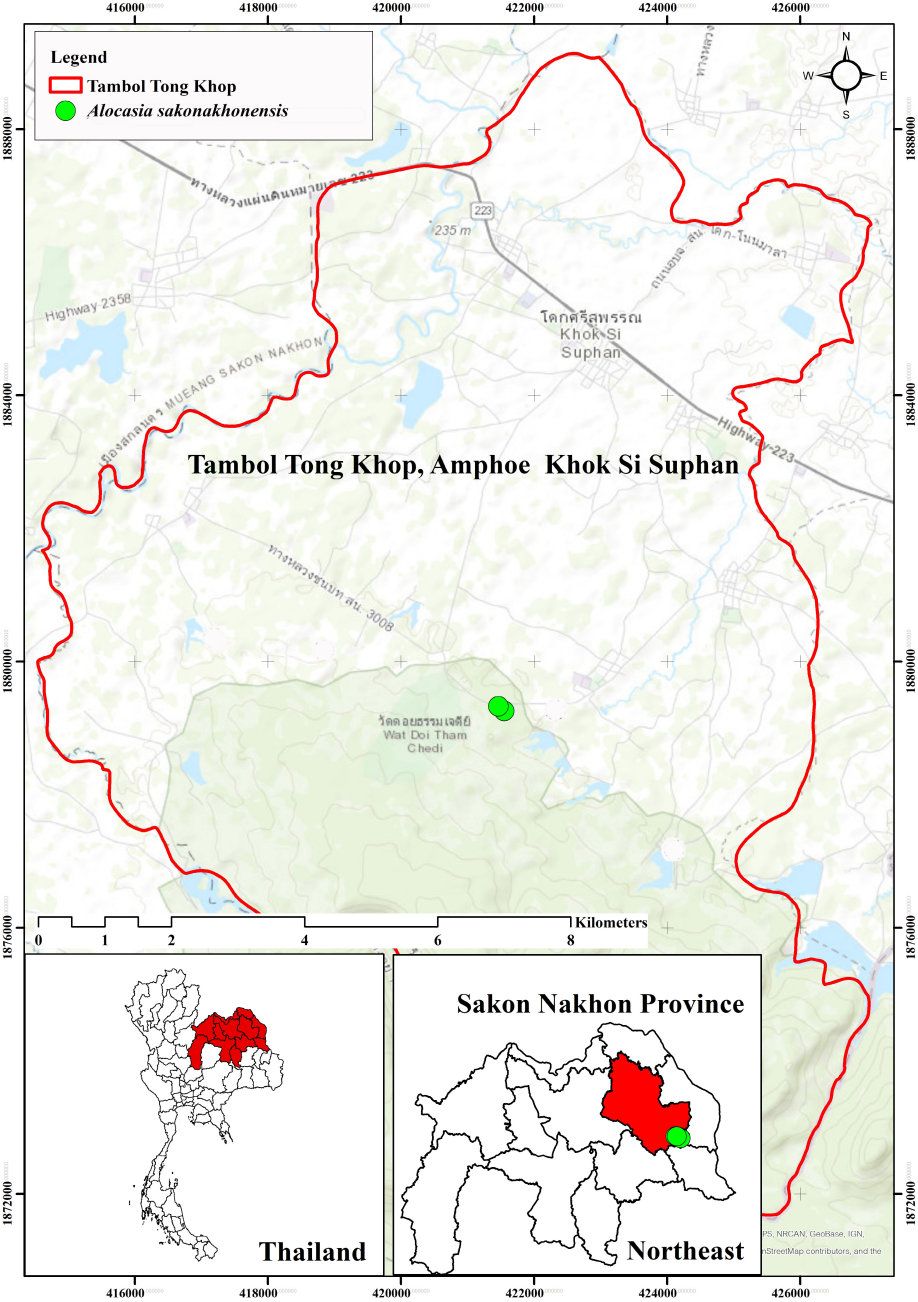
Distribution of *Alocasia sakonakhonensis* Chatan & Promprom (shown by green) in Sakon Nakhon Province, Thailand.

Until now, *Alocasia sakonakhonensis* Chatan & Promprom has been considered endemic to Thailand. It has been found in only one population in the Phu Phan mountain range, located in Sakon Nakhon province, northeastern Thailand. (Figure 3).

### 3.6 Ecology

This new species grows in shaded areas in dry evergreen forests at an elevation of 300–320 m. The plants grow in moist places on limestone, especially along streams.

### 3.7 Vernacular name

Bon Pa.

### 3.8 Etymology

The specific epithet of *Alocasia sakonakhonensis* refers to the type locality, the Sakon Nakhon province in the northeast of Thailand.

### 3.9 Preliminary conservation status

*Alocasia sakonakhonensis* has not been recorded or described so far. This species grew in two small populations under the forest canopy in Sakon Nakhon province at an altitude of 300–380 m. The plant is estimated to have fewer than 2500 mature individuals, with each subpopulation consisting of fewer than 250 mature individuals. Therefore, it should be considered as “Endangered (EN),” according to IUCN criteria C(C2ai) (IUCN Standards and Petitions Committee, 2024).

### 3.10 Taxonomic notes

*Alocasia sakonakhonensis* represents a non‐pachycaul herbaceous taxon. It shares vegetative similarities with *A. acuminata*, characterized by non‐seasonal dormancy, the absence of pachycaul and basal branching, short internodes, and peltate, bright green leaf blades. However, in terms of morphology, the new species can be readily distinguished from the latter. This distinction is evident in the moderately green color of the petiole and peduncle, which feature numerous dirty mottled dark brown lines or areas measuring 1–3 mm (as opposed to the bright green color without such lines). Additionally, the length of these organs—including the peduncle, spathe, spathe limb, spadix, sterile interstice, and appendix—differs in *A. sakonakhonensis*, being more extended than those found in *A. acuminata* (measuring 25–28 cm, 13.0–14.5 cm, 9.5–10.5 cm, 11–12 cm, 23–25 cm, and 5.0–5.2 cm in length, respectively). The stigma of *A. sakonakhonensis* is distinctly 2–4 lobed, very rarely unlobed (in contrast to *A. acuminata*, which is very slightly unlobed). Furthermore, the petiolar sheaths of *A. sakonakhonensis* are open, unlike those of *A. acuminata*, which are closed. In *A. acuminata*, the appendix exhibits a dull yellow‐brown color (compared to the white color in *A. acuminata*). Additional details regarding the morphological distinctions between *A. sakonakhonensis* and *A. acuminata* are presented in Table 1. Below is a modified key to the non‐pachycaul herbaceous taxa of *Alocasia* in Thailand, adapted from Boyce and Sookchaloem (2012).

**TABLE 1:** Comparison of the different morphological characters between *Alocasia sakonakhonensis* Chatan & Promprom Chatan & Promprom and *Alocasia acuminata* Schott.

Morphological characters	*Alocasia sakonakhonensis*	*Alocasia acuminata*
Petioles	Moderately green with numerous dirty dark brown 1**–**3 mm lines	Bright green without numerous dirty dark brown 1**–**3 mm lines
Lamina	Peltate for 37%–43% of their length; anterior lobe apex cuspidate	Peltate for 25%–30% of their length, anterior lobes acute
Peduncle	25–28 cm long, moderately green with numerous dirty dark brown 1**–**3 mm lines or areas	9–20 cm long, green without numerous dirty dark brown 1**–**3 mm lines
Spathe	13.0–14.5 cm long, moderately constricted ca. 3.5–4.0 cm from the base lower part of spathe bright green; limb 9.5–10.5 cm long	7–10 cm long, moderately constricted ca. 1.5–2.5 cm from the base; lower spathe green; limb 5.5–7.5 cm long
Spadix	ca. 11–12 cm long	ca. 6–9.5 cm long
Ovary	Globose or slightly 3‐lobed‐globose or depressed globose or slightly elliptic, 2–3 mm diam., light green; style 1.2–1.5 mm long; stigma off‐white with a hint of yellow or strong pale yellow, distinctly 2–4 lobed or rarely unlobed	Ovaries subglobose, ca. 1.5–2 mm diam.; stigma subsessile, white, unlobed or very slightly lobed
Sterile interstice	23–25 mm	7–10 mm
Synandria	4–8‐merous, more or less hexagonal to circular, 1.5–4.0 mm diam.	4–6‐merous, more or less hexagonal, ca. 2 mm diam.
Appendix	5.0–5.2 cm long, slightly narrower than male flower zone, cylindrical lower part ca. 2/3, elongate‐conic upper part ca. 1/3, dull yellow‐brown	2.5–3.5 cm long, approximately as thick as male flower zone, elongate‐conic, white

Key to non‐pachycaul herb taxa of *Alocasia* in Thailand.

**Table:** 

1	Plant with seasonally dormancy period; leaf blade not peltate; plant producing long (up to 11 cm) horizontal or spreading stolon	*A. hypnosa*
1	Plant almost without seasonally dormancy period, leaf blade usually peltate in sinus, plant without long stolons	2
2	Stem erect and basally much branched; herb only known from areas of human disturbance	*A. cucullata*
2	Stem erect to decumbent, not branching basally; herb from national forest	3
3	Internode conspicuously elongated; each leaf subtended by a conspicuous cataphyll	4
3	Internode inconspicuously elongated; each leaf not noticeably interspersed with cataphylls	5
4	Petioles purple‐brown to pink or green, strikingly obliquely mottled chocolate brown; leaf blade thinly papery, posterior lobes free or only partially fused.	*A. longiloba*
4	Petioles green, unmarked; leaf blade coriaceous or highly coriaceous and subsucculent, posterior lobes joined for almost their entire length (leaf peltate)	*A. perakensis*
5	Petioles and peduncles moderately green with numerous dirty mottled dark brown 1–3 mm lines or areas; sterile interstice more than 15 mm long; stigma distinctly 2–4 lobed or rarely unlobed	*A. sakonakhonensis*
5	Petioles and peduncles bright green without dirty mottled dark brown 1–3 mm lines or areas; sterile interstice less than 15 mm long; stigma unlobed or very slightly lobed	*A. acuminata*

## AUTHOR CONTRIBUTIONS

**Wannachai Chatan:** Conceptualization (lead); data curation (equal); funding acquisition (equal); investigation (equal); methodology (equal); project administration (equal); writing – original draft (lead); writing – review and editing (lead). **Wilawan Promprom:** Data curation (equal); formal analysis (equal); funding acquisition (equal); investigation (equal); validation (equal); visualization (equal). **Phukphon Munglue:** Data curation (equal); formal analysis (equal); methodology (equal).

## FUNDING INFORMATION

This research project was financially supported by Thailand Science Research and Innovation (TSRI) (FF‐6606672/2566).

## CONFLICT OF INTEREST STATEMENT

The authors declare no conflicts of interest.

## Data Availability

There is no other data declaration.
